# Targeted and Untargeted Metabolomics to Explore the Bioavailability of the Secoiridoids from a Seed/Fruit Extract (*Fraxinus angustifolia* Vahl) in Human Healthy Volunteers: A Preliminary Study

**DOI:** 10.3390/molecules201219845

**Published:** 2015-12-11

**Authors:** Rocío García-Villalba, Francisco A. Tomás-Barberán, Pascale Fança-Berthon, Marc Roller, Pilar Zafrilla, Nicolas Issaly, María-Teresa García-Conesa

**Affiliations:** 1 Research Group on Quality, Safety and Bioactivity of Plant Foods, Department Food Science and Technology, Centro de Edafología y Biología Aplicada del Segura (CEBAS)–Consejo Superior de Investigaciones Científicas (CSIC), P. O. Box 164, Campus de Espinardo, Murcia 30100, Spain; rgvillalba@cebas.csic.es (R.G.-V.); fatomas@cebas.csic.es (F.A.T.-B.); 2 Naturex SA, Site d’AgroParc, BP 1218, 84911 Avignon Cedex 9, France; p.fancaberthon@naturex.com (P.F.-B.); m.roller@naturex.com (M.R.); 3 Department of Food Technology and Nutrition, Catholic University of San Antonio, Campus de los Jerónimos, N° 135, Guadalupe, Murcia 30107, Spain; mpzafrilla@ucam.edu; 4 Naturex Spain SL, Autovia A3, Salida343, Camino de Torrent s/n, Quart de Poblet, Valencia 46930, Spain; n.issaly@naturex.com

**Keywords:** tyrosol, ligstroside-aglycone, UPLC-ESI-QTOF, absorption, metabolism, phase II conjugation

## Abstract

The bark, seeds, fruits and leaves of the genus *Fraxinus* (Oleaceae) which contain a wide range of phytochemicals, mostly secoiridoid glucosides, have been widely used in folk medicine against a number of ailments, yet little is known about the metabolism and uptake of the major *Fraxinus* components. The aim of this work was to advance in the knowledge on the bioavailability of the secoiridoids present in a *Fraxinus angustifolia* Vahl seed/fruit extract using both targeted and untargeted metabolomic analyses. Plasma and urine samples from nine healthy volunteers were taken at specific time intervals following the intake of the extract and analyzed by UPLC-ESI-QTOF. Predicted metabolites such as tyrosol and ligstroside-aglycone glucuronides and sulfates were detected at low intensity. These compounds reached peak plasma levels 2 h after the intake and exhibited high variability among the participants. The ligstroside-aglycone conjugates may be considered as potential biomarkers of the *Fraxinus* secoiridoids intake. Using the untargeted approach we additionally detected phenolic conjugates identified as ferulic acid and caffeic acid sulfates, as well as hydroxybenzyl and hydroxyphenylacetaldehyde sulfate derivatives which support further metabolism of the secoiridoids by phase I and (or) microbial enzymes. Overall, the results of this study suggest low uptake of intact secoiridoids from a *Fraxinus angustifolia* Vahl extract in healthy human volunteers and metabolic conversion by esterases, glycosidases, and phase II sulfo- and glucuronosyl transferases to form smaller conjugated derivatives.

## 1. Introduction

**Figure 1:**
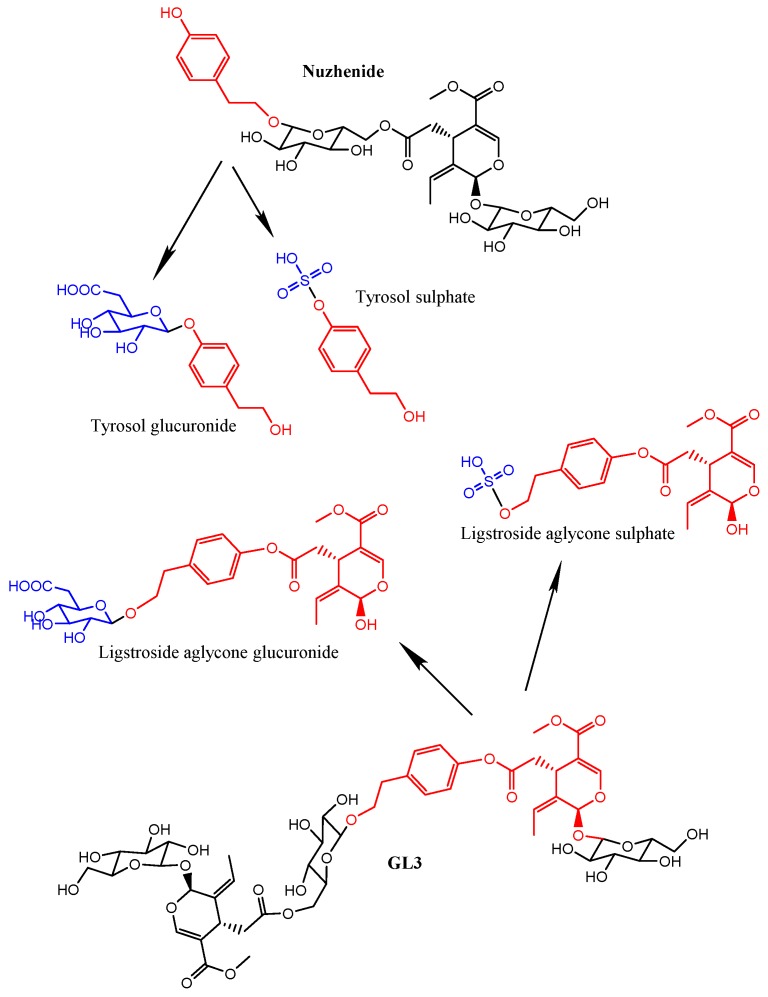
Chemical structures of the main secoiridoid constituents (nuzhenide and GL3) found in the *Fraxinus* *angustifolia* Vahl extract and of the specific metabolites detected in plasma and urine after the intake. The fragments originated from the hydrolysis of the secoiridoids are marked in red (tyrosol and ligstroside-aglycone). The derivatives produced by phase II conjugation are shown in blue color.

The genus *Fraxinus* (Oleaceae) which contains a wide range of phytochemicals, mostly secoiridoid glucosides, coumarins and phenylethanoids, but also some flavonoids, benzoquinones, indole derivatives and simple phenolics has been commonly used in traditional medicine against various inflammatory diseases, infections, constipation, as a diuretic and as a hepatoprotective agent [1]. Additionally, an extract produced from the seeds/fruits of *Fraxinus* has been shown to moderate body weight gain, body fat, glucose and insulin levels, fatty liver incidence as well as blood pressure in various animal models of obesity, diabetes and hypertension [2,3,4,5] supporting potential beneficial properties of *Fraxinus* against metabolic disorders. These beneficial effects were observed with doses ranging between 100 and 200 mg per kg of body weight per day, corresponding to a Human Equivalent Dose (HED = animal dose in mg/kg × (animal weight in kg/human weight in kg)^0.33^) of ~1.0 g/day for a 60 kg person. Notably, the acute administration of 1 g of the *Fraxinus* extract first evidenced the reduction of glycemia in healthy humans [6] and the daily administration of 1 g of the *Fraxinus* extract for three weeks in elderly overweight/obese subjects resulted in a significantly lower incremental glucose area under the curve, lower 2 h blood glucose levels and modified levels of fructosamine and adiponectin:leptin ratio [7]. This *Fraxinus* extract, prepared using a patented extraction protocol [8], was reported to contain a standardized amount (~10%) of the secoiridoid glycoside nuzhenide (CAS registry number 60037-39-0) plus the dimeric secoiridoid glycoside GL3 (glycosylated esters of tyrosol and elenolic acid; CAS registry number 39011-92-2) (Figure 1). These two compounds were shown to activate peroxisome proliferator-activated receptor alpha (PPARα) and to inhibit adipocyte differentiation in cultured adipocytes providing a potential molecular mechanism underlying the metabolic regulatory activity of the *Fraxinus* extract [9]. These secoiridoids have thus been proposed as the main bioactive constituents of the extract potentially responsible for its beneficial effects [4,6,9] but the biological effects of these compounds within the organism depend largely on their metabolism and uptake from the gastrointestinal tract. 

The bioavailability of other secoiridoids from olive oil [10,11,12] and olive leaf extracts [13,14,15,16] that belong to the same *Oleaceae* family as *Fraxinus* have been reported but, to date and to the best of our knowledge, there was no information on the bioavailability, the type of metabolites formed and the pharmacokinetics of nuzhenide, GL3 and other secoiridoids present in the *Fraxinus* extract. The characterization of *in vivo* circulating metabolites derived from the intake of plant dietary phytochemicals remains an essential step for the identification of the actual bioactive molecules and for the understanding of the mechanisms underlying the beneficial properties of these plant compounds. However, unravelling the metabolic conversion of plant dietary bioactive compounds and identifying their derived metabolites in humans remains an intricate and challenging task. Metabolomics study global changes in the entire metabolite set of certain cells, tissues, organs and organisms. It combines strategies to identify and quantify metabolites using sophisticated analytical technologies (different chromatographic separation techniques, such as liquid or gas chromatography coupled to mass spectrometry detection system) with the application of statistical and multi-variate methods for the extraction of information and data interpretation. The application of metabolomics in food systems (called as “food metabolomics” or “foodomics”) is a very powerful technology to further progress in the elucidation and understanding of the metabolism and absorption of dietary compounds [17]. By providing highly sensitive and specific metabolic profiles in body fluids and tissues, it allows for the identification of potential bioactive metabolites and biomarkers of exposure to diet compounds [18]. Two ways of identifying metabolites have been developed: (i) the targeted approach which involves the selection of specific candidates that are likely to appear in the biological samples [19] and (ii) the untargeted approach that uses advanced analytical tools to extract unknown information of the metabolome from large data sets [20,21,22].

The aim of this pilot study was to investigate the metabolic fate of the main secoiridoids present in a *Fraxinus* *angustifolia* Vahl seed/fruit extract in a group of healthy volunteers. Using an UPLC-ESI-QTOF-MS, we searched for specific anticipated metabolites (targeted metabolomics) as well as for other non-predicted metabolites (untargeted metabolomics) that appear in the urine and plasma of the volunteers following the consumption of 1 g of the extract.

## 2. Results and Discussion

### 2.1. Fraxinus Extract Content in Secoiridoids

**Figure 2:**
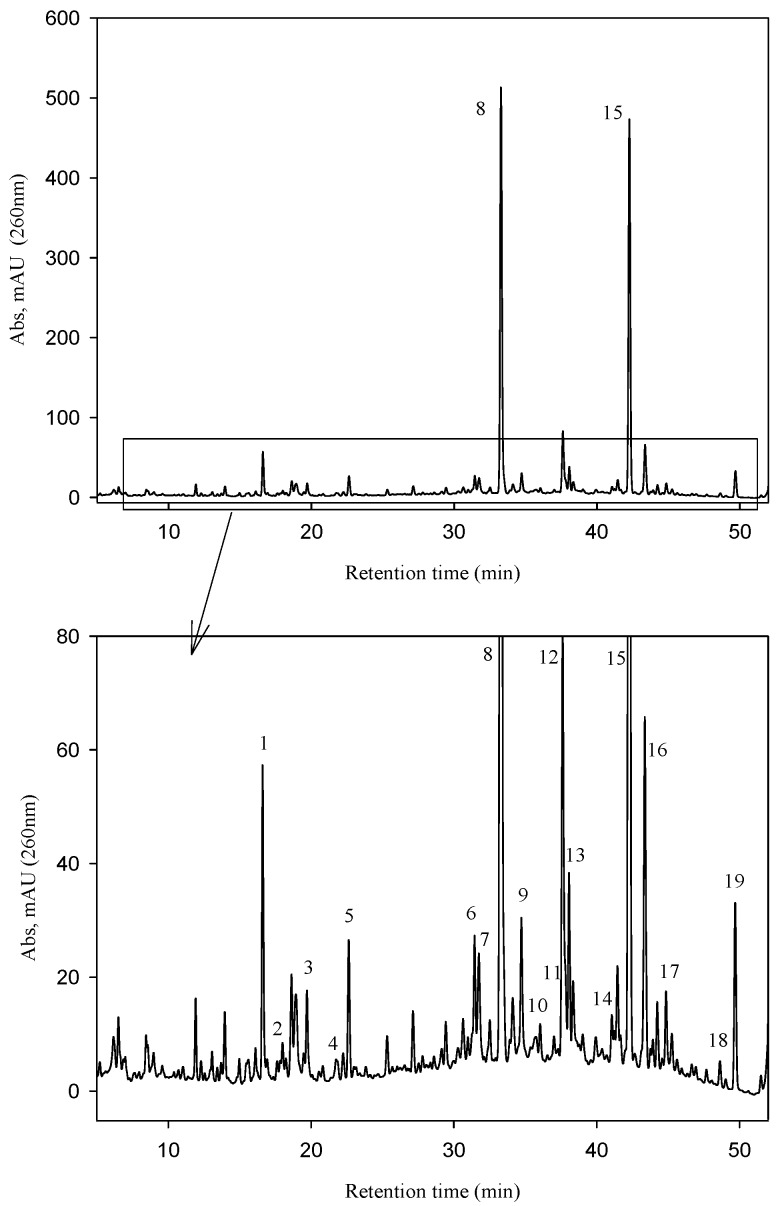
HPLC-UV (260 nm) chromatogram of the *Fraxinus angustifolia* Vahl seed/fruit extract used in the study.

The UV chromatographic profile of the phenolic compounds extracted from the *Fraxinus* *angustifolia* extract used in this study is shown in Figure 2. We confirmed that the two major polyphenolic components, the secoiridoid glucosides nuzhenide (peak 8) and GL3 (peak 15), were present and amounted up to 5.92% ± 0.14% and 4.75% ± 0.09%, respectively (total quantity: 10.67% ± 0.23%). Various other compounds were also detected in the extract based on their mass [M − H]^−^, the MS/MS fragmentation pattern and data available in the literature (Table 1) [1,4]. Most of them were secoiridoid glucosides such as salidroside, oleoside, oleoside-11-methyl ester, glucosylformoside, excelside B, GL5 and other isomers of nuzhenide and GL3. These compounds contain as part of their molecular structure: tyrosol (4-hydroxyphenyl ethylalcohol), elenolic acid and (or) elenolic acid linked to tyrosol (ligstroside-aglycone) (Figure 1). Other secoiridoids like nuzhenide 11-methyl oleoside (isomer of GL3) and nuzhenide di(11-methyloleoside), previously described in olive seeds [23], were first time tentatively identified in the *Fraxinus* extract. We also detected various compounds displaying a UV spectrum similar to that typical of hydroxycinnamic acid as well as verbascoside (ester of hydroxytyrosol, caffeic acid and the sugar α-l-rhamnopyranosyl-(1-3)-β-d-glucopyranose).

To understand the potential metabolic effects of the compounds present in the *Fraxinus* extract we need to determine their bioavailability and thus, we next investigated the bioavailability of the main secoiridoid glucosides identified in the extract using a targeted approach.

**Table 1:** Compounds identified in the *Fraxinus angustifolia* extract used in this study.

Peak	Compounds	Rt (UV)	[M − H]^−^	MS/MS
1	Salidroside	16.60	299	179,119
2	Hydroxycinnamic acid derivative	16.92	487	295, 179, 135
3	Oleoside	19.70	389	371, 345, 209, 179
4	Oleoside 11-methyl ester	22.63	403	223, 179
5	Hydroxycinnamic acid derivative	21.75	369	207, 192, 179
6	Verbascoside	31.45	623	461, 315, 251
7	Nuzhenide isomer	31.75	685	523, 453, 421, 299, 223
8	Nuzhenide	33.28	685	523, 453, 421, 299, 223
9	Nuzhenide isomer	34.72	685	523, 453, 421, 299, 223
10	1-*O*-β-d-Glucosylformoside	36.03	685	523, 453, 385, 299, 223
11	Nuzhenide derivative	37.01	727	685, 565, 523, 453, 341, 299
12	Excelside B	37.61	685	565, 361, 291, 260
13	GL3 isomer *	41.06	1071	909, 685, 523, 385
14	GL3 isomer *	41.45	1071	909, 771, 685, 609, 421
15	GL3	42.20	1071	909, 771, 685, 453, 385
16	GL3 isomer *	43.36	1071	909, 685, 478, 361
17	Nuzhenide di (11-methyloleoside)	44.96	1457	1157, 1071, 934, 771, 685
18	GL5 isomer	48.70	909	771, 747, 523, 361, 259
19	GL5 isomer	49.77	909	771, 747, 645, 523, 361

* Some of GL3 isomers could correspond to nuzhenide 11-methyloleoside.

### 2.2. Fraxinus Extract Derived Specific Plasma and Urine Metabolites: Targeted Analysis

The targeted approach involves searching for a selection of predicted metabolites that are likely to appear in plasma and urine. Although scarce, data about absorption and metabolism of oleuropein, a secoiridoid glycoside (ester of hydroxytyrosol and elenolic acid linked to glucose) present in olive leaf extracts, were useful to develop the targeted analysis. It has been reported that oleuropein was absorbed in very small quantities as such and also conjugated as glucuronide [13,14,24]. The predominant circulating metabolites were oleuropein-aglycone derivatives and the conjugates (glucuronide, sulfate and methylated) of hydroxytyrosol suggesting extensive hydrolysis and phase II conjugation of the parent compound [13,14,15,16]. Similar metabolites were observed after the intake of olive oil, a foodstuff rich in oleuropein-aglycone [10,11]. Based on these results, we specifically searched for a range of metabolites (Supplementary Table S1) covering the parent original compounds, their aglycones, a number of potential fragments and the corresponding phase II derivatives of all of them. None of the secoiridoid glucosides present in the *Fraxinus* extract were detected in the urine and plasma samples indicating that the parent original compounds may not be directly absorbed and must undergo some metabolic conversion. Similarly to the case of oleuropein, we found that the main circulating metabolites following the intake of the *Fraxinus* extract were the conjugates of the hydrolysis products of the parent compounds: a glucuronide and two sulfate conjugates of tyrosol as well as a glucuronide and a sulfate conjugate of the ligstroside-aglycone (Table 2). The molecular formulae of these metabolites were obtained with high score (>91) and low error (<3.8 ppm). MS/MS spectra of tyrosol conjugates showed a characteristic fragment at *m*/*z* 137.0606 indicative of a tyrosol moiety while a fragment ion at *m*/*z* 361.1288 representative of a ligstroside-aglycone moiety appeared in the ligstroside-aglycone conjugates. Fragments at *m*/*z* 79.9574 and 175.0246 were indicative of sulfate and glucuronic acid moiety respectively.

**Table 2:** Targeted analysis: metabolites detected in the urine and plasma samples of the volunteers following the consumption of the *Fraxinus angustifolia* extract. All the metabolites were present at low intensity.

Compounds	Retention Time (min)	*m*/*z* Experimental	Score	Error	Molecular Formulae	MS/MS Fragments
Tyrosol glucuronide	5.30	313.0937	91.75	−3.13	C_14_H_18_O_8_	137.0608, 175.0247
Tyrosol sulfate 1	5.79	217.0168	96.09	3.80	C_8_H_10_O_5_S	137.0604, 79.9572
Tyrosol sulfate 2	13.00	217.0174	95.35	1.78	C_8_H_10_O_5_S	137.0606, 79.9574
Ligstroside-aglycone * glucuronide	14.66	537.1613	97.9	−0.05	C_25_H_30_O_13_	361.1288, 175.0246
Ligstroside-aglycone * sulfate	15.02	441.0868	95.6	−1.74	C_19_H_22_O_10_S	361.1295, 79.9577

* Ligstroside-aglycone (elenolic acid-tyrosol).

**Figure 3:**
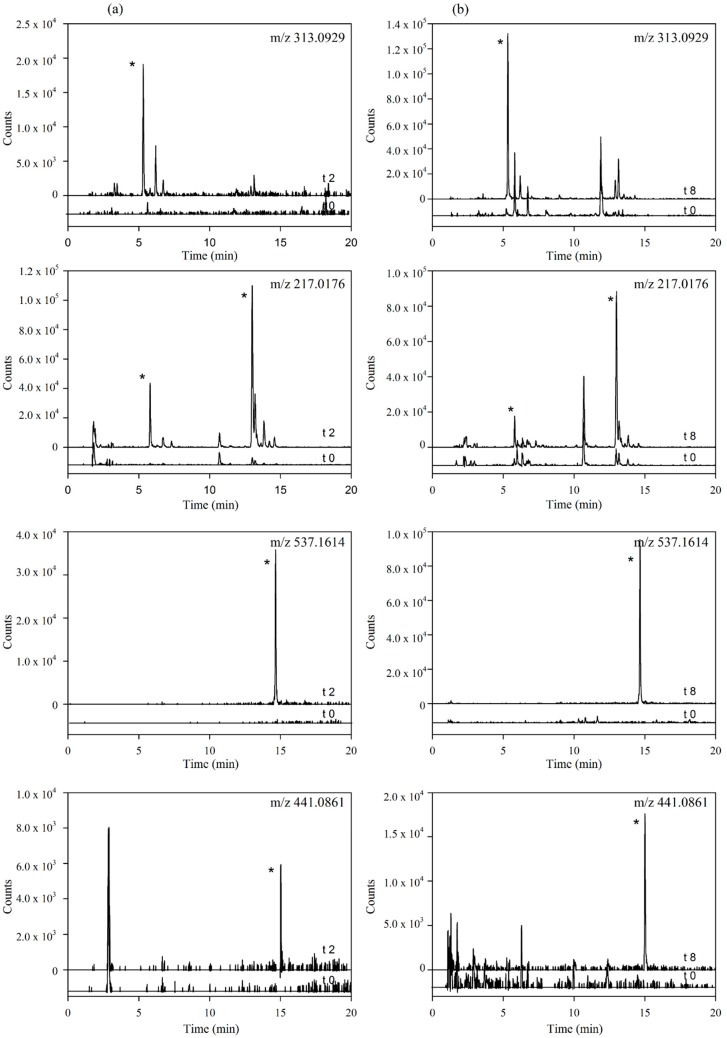
Extracted Ion Chromatograms (EICs) of the main targeted metabolites identified in (**a**) plasma samples (time 0 *vs.* 2 h) and (**b**) urine samples (time 0 *vs.* 8 h). (* indicate peaks corresponding to the metabolites identified for each mass in Table 2).

These results provide the first evidence of the hydrolysis of the secoiridoids nuzhenide and GL3 and possibly of other minor secoiridoids (salidroside, glucosylformoside, excelside A and B, GL3 and GL5 isomers) present in the *Fraxinus* extract via the loss of glucose moieties (Figure 1). This hydrolysis might be mediated by the host and (or) microbial glycosidases and (or) esterases [25,26] followed by phase II glucuronidation or sulfation of the aglycone molecules. The extracted ion chromatograms (EICs) of the four metabolites in plasma samples at time 0 and 2 h and in urine samples at 0 and 8 h are further illustrated in Figure 3.

**Figure 4:**
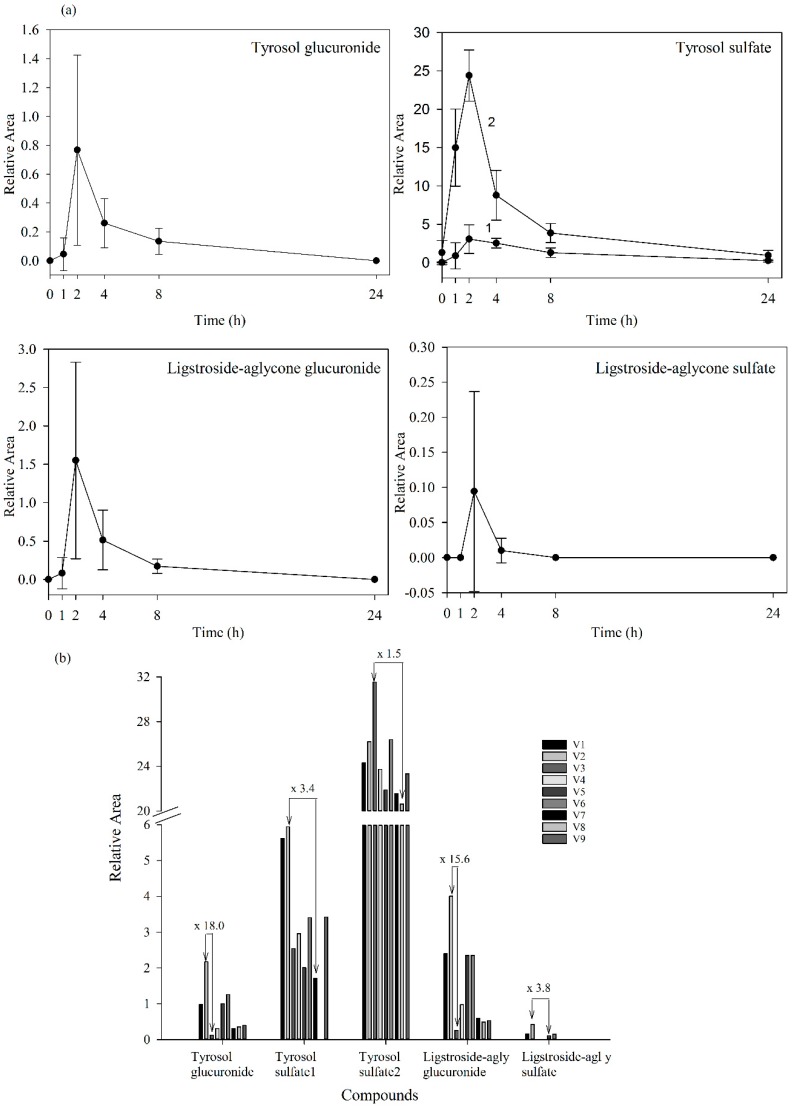
(**a**) Plasma levels-time courses of the targeted metabolites detected. Values are means of nine volunteers with SD shown by vertical bars (1 and 2 correspond to two isomers of tyrosol sulfate); (**b**) Inter-individual variability in the content of the targeted metabolites observed in plasma 2 h after the consumption of the *Fraxinus angustifolia* Vahl extract. Differences between maximum and minimum values are indicated. All results are shown as relative peak areas (relative area = (peak area metabolite × 100/peak area internal standard)).

**Figure 5:**
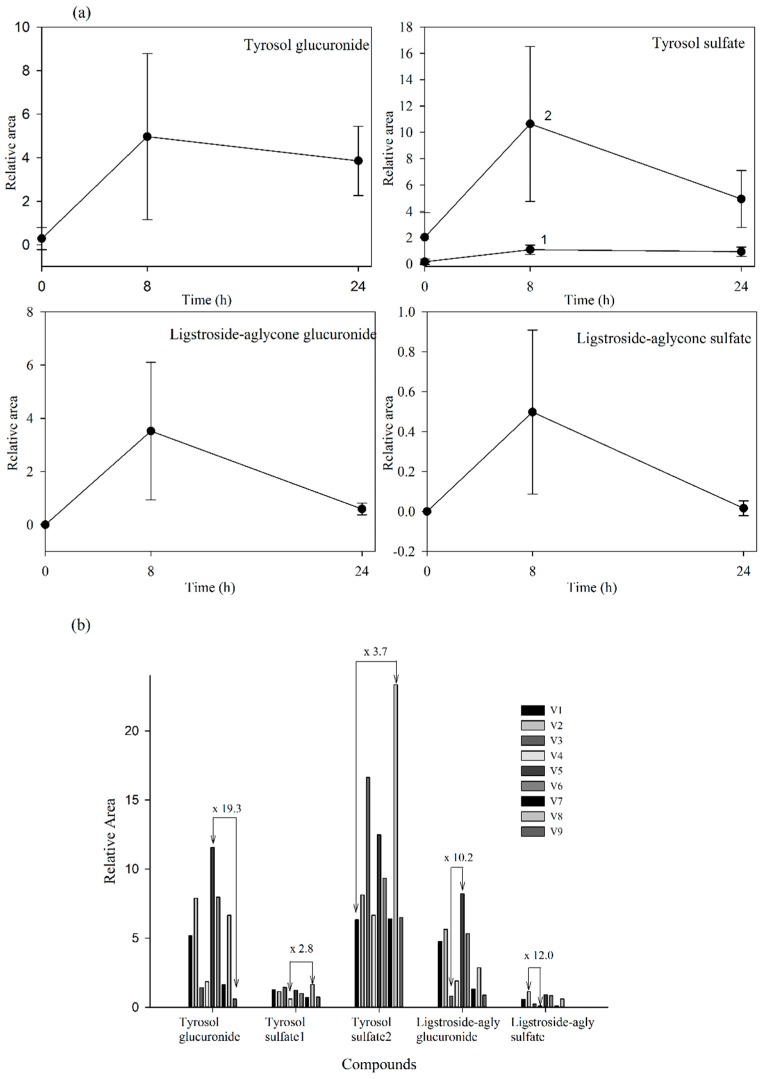
(**a**) Urine concentration-time courses of the targeted metabolites detected. Values are means of nine volunteers with SD shown by vertical bars (1 and 2 correspond to two isomers of tyrosol sulfate); (**b**) Inter-individual variability in the content of the targeted metabolites observed in urine collected during the first 8 h after the consumption of the *Fraxinus angustofolia* Vahl extract. Differences between maximum and minimum values are shown. All results are shown as peak areas (relative area = (peak area metabolite × 100/peak area internal standard)).

Since standards for these compounds were not available, we were not able to accurately quantify the levels of the detected metabolites. However, using relative peak areas (relative area = (peak area metabolite × 100/peak area IS)) we were able to plot the plasma and urine pharmacokinetic behavior of each metabolite. Compounds identified in urine were quantified after adjustment for the volume of urine produced. The absorption-time profiles in plasma and excretion-time profiles in urine for each metabolite are shown in Figure 4a and Figure 5a, respectively (mean values ± SD).

Our results evidenced that the secoiridoid metabolites derived from the *Fraxinus angustifolia* extract reached their maximum levels in plasma 2 h after the intake, *i.e.*, slightly delayed in comparison with the maximum peak concentration for hydroxytyrosol and oleuropein conjugates (within the 35–75 min post-ingestion of olive leaf extract) [13,16]. The tyrosol sulfates were still detected in plasma at 24 h whereas the ligstroside-aglycone sulfate was not detected any longer after 4 h. In urine, all the metabolites examined were detected mostly at 8 h after the intake of the extract, similarly to the excretion of hydroxytyrosol and oleuropein conjugates [13,16]. Some metabolites, especially those derived from tyrosol, continued appearing at 24 h. As it is the case for other dietary polyphenols [27], the levels of all the examined metabolites exhibited a high inter-individual variability as depicted in Figure 4b and Figure 5b (individual values for each 2 h-plasma and 8 h-urine metabolite after the intake of the *Fraxinus* extract, respectively). Differences between the maximum and minimum levels of metabolites were up to 18-fold and 15-fold in plasma and 19-fold and 10-fold in urine for tyrosol glucuronide and ligstroside-aglycone glucuronide, respectively. Generally, the translation of beneficial metabolic effects of dietary polyphenols from pre-clinical animal models to human intervention studies has shown inconsistent evidences possibly and partly due to the variability in the metabolism and uptake of these compounds [28]. In the case of the *Fraxinus* extract which has been shown to moderate fasting blood glucose (FBG) and insulin levels in rodent models of obesity, intervention in obese and overweight subjects resulted in 2 h-blood glucose improvement following an oral glucose tolerance test but not significant differences for FBG and insulin levels were observed [7]. Small sample size and variability in the capacity of each volunteer to absorb and metabolize the secoiridoids from the *Fraxinus* extract may contribute to explain the lack of current significant mean effects in humans for those two metabolic biomarkers. Other factors such as gender can influence the bioavailability of the secoiridoids as previously suggested for olive leaf extracts [13] but a difference between male and female was not apparent in this study. The variability in the bioavailability of the secoiridoids from the *Fraxinus* extract and the factors affecting it needs to be further and critically assessed.

### 2.3. Untargeted Approach to Study Non-Predicted Secoiridoid Metabolites Appearing in Plasma and Urine Samples

**Figure 6:**
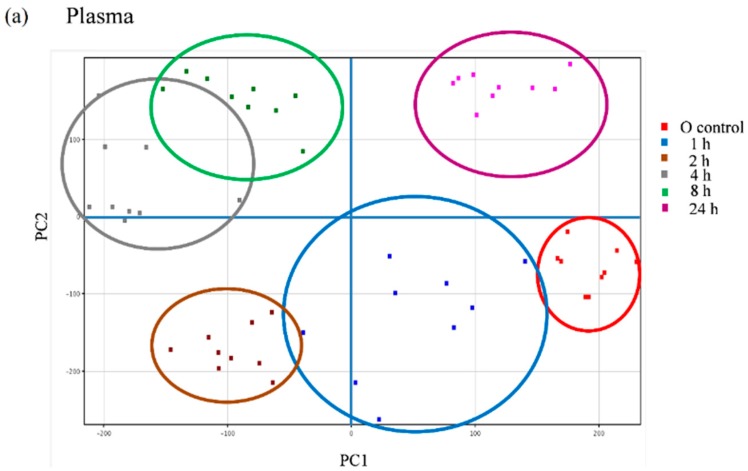

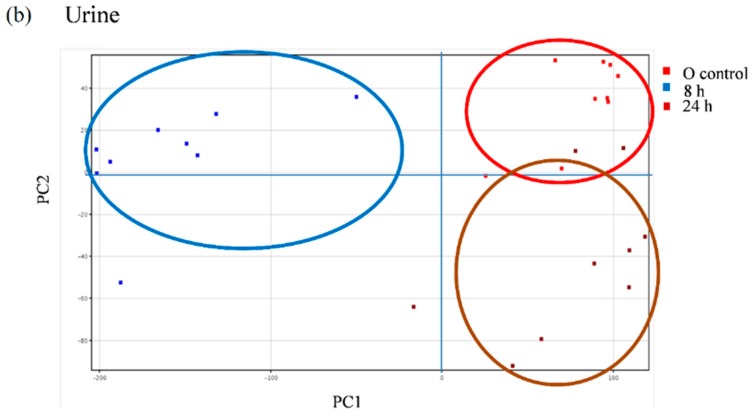
Principal component analysis (PCA) scores plot of the plasma (**a**) and urine (**b**) samples of nine volunteers analyzed at baseline (control) and at different time-points after the intake of the *Fraxinus* *angustifolia* Vahl extract.

We further explored the appearance of other non-predicted potential metabolites directly derived from the metabolic transformation of the secoiridoids in the plasma and urine samples of the volunteers following the intake of the *Fraxinus* extract using an untargeted strategy that enables the extraction of the required information from large data sets. Between 600 and 800 peaks in the plasma samples and 500 and 2000 peaks in the urine samples were extracted by chromatographic deconvolution. After alignment based on mass accuracy and retention time, 2360 unique entities in plasma and 6216 in urine were identified. Filtering by frequency (data present in at least 50% of the volunteers in at least one time-point) reduced the number of molecular features (MFs) to 681 in plasma and 1243 in urine. The filtered features were then compared between the baseline samples and the different time-points examined using one-way ANOVA yielding a total of 98 significantly different features in plasma and 336 in urine (*p* < 0.05, fold-change cut-off = 2.0). Unsupervised PCA was used to visualize these results into six well-discriminated groups in plasma (Figure 6a) and three groups in urine (Figure 6b). The baseline samples resulted clearly separated from those samples taken at the different time-points.

The metabolites responsible for the discrimination of these time-points groups may be: (i) of exogenous origin, *i.e.*, resulting from the absorption and metabolism of *Fraxinus* compounds but also from other compounds taken with the diet and (ii) of endogenous origin, *i.e.*, metabolites that could be altered by the intake of the *Fraxinus* extract [20]. We concentrated our efforts towards the identification of those metabolites that were significantly increased following the ingestion of the extract and that may derive directly from the secoiridoids present in it. A total of 50 MFs in the plasma samples and 52 MFs in the urine samples were found consistently at higher levels in the post-ingestion samples than in the baseline samples. Using their exact mass and MS/MS fragments we were able to tentatively identify seven compounds in the plasma and four compounds in the urine (Table 3) that showed high p(corr) values. Tyrosol sulfate in plasma and ligstroside-aglycone glucuronide both in plasma and urine, previously detected in the targeted analysis, were confirmed as significantly different offering partial validation of the untargeted approach. These results support that ligstroside-aglycone glucuronide may constitute a good candidate as a “biomarker of intake” for the *Fraxinus* extract since this metabolite is specifically originated from the secoiridoids present in the extract, was not present in the urine and plasma samples at baseline and was identified as one of the MFs that contribute to differentiate between the post ingestion time-point samples and the baseline samples (p(corr) = 0.00275 in plasma and 0.0108 in urine). We additionally detected the phenolic derivatives, ferulic acid and caffeic acid sulfate. Further, and in support of microbial metabolism of the *Fraxinus* secoiridoids, we were able to detect a significant rise in plasma and/or urine of the sulfate conjugates of the microbial metabolites 4-hydroxybenzyl alcohol, 4-hydroxyphenylacetaldehyde and 3,4-dihydroxyphenylacetaldehyde (DOPAL). These metabolites may result from the fermentation of tyrosol and (or) hydroxytyrosol by fecal microbial metabolism [29,30]. These metabolites may derive from the fermentation of different dietary polyphenols and thus we cannot unequivocally relate them to the intake of the *Fraxinus* secoiridoids.

The main objective of this study was to contribute to identify some of the metabolites circulating *in vivo* in humans following the intake of a *Fraxinus angustifolia* extract and that could be potentially involved in the metabolic regulatory effects attributed to this natural extract. We acknowledge that this is a preliminary study with a small number of volunteers and that future studies should include a larger number of participants to account for the variability in the human absorption and metabolism of plant bioactive compounds. Nevertheless we have shown that tyrosol conjugates constitute some of the main metabolites circulating in plasma after the intake of the extract. Since it has been reported that tyrosol moderates the levels of blood glucose and insulin in streptozotocin-induced diabetic rats [31] it is possible that the tyrosol conjugates may be partially involved in the glycemic regulatory effects associated with the consumption of the *Fraxinus* extract [2,3,4,6,7].

**Table 3:** Metabolites significantly increased in the plasma and urine samples of the volunteers following the intake of *Fraxinus* extract and related to the family of phenolic compounds: untargeted approach.

Exact Mass	Retention Time (min)	Error (ppm)	Score	MS/MS	Molecular Formula	Tentatively Identified Metabolite	Time-Points (h) *	p(corr)
Plasma								
259.9985	8.28	0.02	98.12	179.0352, 135.0455, 96.9592	C_9_H_8_O_7_S	Caffeic acid sulfate	1, 2, 4, 8	3.85 × 10^−8^
274.014	8.58	1.92	98.53	193.0505	C_10_H_10_O_7_S	Ferulic acid sulfate	2, 4	1.70 × 10^−3^
204.0087	10.41	3.98	94.87	123.0449, 79.9575	C_7_H_8_O_5_S	4-hydroxybenzyl alcohol sulfate	1 ,2, 4	3.50 × 10^−6^
274.0142	10.98	2.01	97.79	193.0505	C_10_H_10_O_7_S	Ferulic acid sulfate	1, 2	1.04 × 10^−15^
216.0087	12.20	1.55	98.84	135.0047	C_8_H_8_O_5_S	4-hydroxyphenylacetaldehyde sulfate	2, 4	9.55 × 10^−6^
218.024	13.01	2.3	97.58	137.0606, 122.0374, 79.9574	C_8_H_10_O_5_S	Tyrosol sulfate	1, 2, 4	1.98 × 10^−12^
538.1688	14.64	−0.02	99.04	493.1712, 401.8835, 361.1288, 175.0246, 153.0917, 113.0240	C_25_H_30_O_13_	Ligstroside-aglycone glucuronide	1, 2, 4, 8	2.75 × 10^−3^
Urine								
232.0048	8.23	−1.6	98.03	203.2221, 151.0399, 108.0213	C_8_H_8_O_6_S	3,4-dihydroxyphenylacetaldehyde (DOPAL)-sulfate *	8, 24	1.25 × 10^−2^
368.1114	8.39	−0.63	98.69	193.0505,134.0376	C_17_H_20_O_9_	Ferulic acid derivative	8, 24	1.98 × 10^−3^
274.0152	10.98	−1.71	97.52	193.0508, 149.0243, 121.0285, 93.0347, 65.0396	C_10_H_10_O_7_S	Ferulic acid sulfate	8, 24	2.24 × 10^−3^
538.1688	14.65	−0.02	99.04	493.1712, 401.8835, 361.1288, 175.0246, 153.0917, 113.0240	C_25_H_30_O_13_	Ligstroside-aglycone glucuronide	8, 24	1.08 × 10^−2^

* Time points in which the metabolites were detected. The time-point exhibiting maximum concentration is underlined.

Our results also show that the main secoiridoids present in the *Fraxinus* extract are not detected in the plasma after the intake of a high dose (1 g) suggesting that these compounds are poorly absorbed and might be retained in the intestine. It is widely known that the food matrix can have a critical impact on the bioavailability of polyphenols [32] and thus, it is plausible that other constituents present in the *Fraxinus* extract such as complex carbohydrates and proteins [8] may affect the absorption of the secoiridoids. We further hypothesize that the secoiridoids may exert some of their metabolic effects at the intestinal level, by modifying the microbiota composition and/or by interacting with the intestinal barrier [28,33]. We cannot discard either that the other constituents of the extract can contribute to the metabolic benefits of *Fraxinus*. These issues require further investigation and point towards a potential prebiotic effect of the *Fraxinus* extract.

## 3. Experimental Section

### 3.1. Chemicals

Pure standards of nuzhenide and GL3 were synthetized and sent to us by Naturex S.A. (Avignon, France). Stock solutions of both compounds were prepared in water (2000 ppm) and standard mixtures were diluted in methanol (MeOH)/water (50/50, *v*/*v*) to a final concentration of 500 ppm. Quercetin (Sigma-Aldrich, St. Louis, MO, USA) was dissolved in MeOH (5000 ppm) and used as internal standard (IS). MeOH and acetonitrile were from J. T. Baker (Deventer, The Netherlands) and formic acid and HCl were from Panreac (Barcelona, Spain). All other chemicals and reagents were of analytical grade. Milli-Q system (Millipore Corp., Bedford, MA, USA) ultrapure water was used throughout the study.

### 3.2. Fraxinus Extract Characterization

The raw material used for the extract preparation was previously identified using a high pressure thin-layer chromatographic method and reported as *Fraxinus excelsior* L. (common ash) [2,3,4,6,7,8,9]. The whole aerial part of the plant containing the seeds has been now re-evaluated combining macroscopic analyses, high pressure thin layer chromatography (HPTLC, CAMAG, Muttenz, Switzerland) and DNA techniques (these analyses were conducted by Ms. Mélissa Feuillatre, Naturex botanist, using authenticated DNA *Fraxinus angustifolia* Vahl fruit as the botanical reference). Further DNA-based authentication by two independent laboratories (AuthenTechnologies LLC, Richmond, CA, USA and DNA Gensee, Le Bourget du Lac, France) has confirmed that the plant material belongs to the specie *Fraxinus angustifolia* Vahl (narrow-leaved ash). The fruits of *Fraxinus angustifolia* Vahl were collected from the Demnat region in central Morocco (by Mr. Ahmed Sghir, Agricultural Engineer). The corresponding voucher was assigned the voucher specimen identifier 1HAB0044#B020/019/A14; Reference AU100815) and is deposited at Naturex Avignon (Avignon, France).

The *F. angustifolia* extract used in this study was prepared from the seeds and fruits, encapsulated (334 mg per capsule) and kindly supplied by Naturex S.A. (Avignon, France, product code EA149251). The full composition of the extract has been previously reported [8]. Briefly, the extract contains a high proportion of carbohydrates (72.4 g/100 g) and small quantities of protein and total dietary fiber (approximately 6 g/100 g each). It also contains relatively high concentrations of glucose and sucrose (3.34 and 3.84 g/100 g, respectively). The extract was standardized to contain approximately 10 % of the secoiridoids, nuzhenide and GL3 using the extraction process described in the patent US 8293292 [34].

The specific phenolic composition of the extract used in this study was monitored using an HPLC-DAD-MS/MS (IT) system. The *Fraxinus* extract (20 mg) was dissolved with 1 mL of MeOH/H_2_O (50/50, *v*/*v*), vortexed for 2 min and ultrasonicated for 10 min prior to centrifugation at 3500× *g* for 10 min. The supernatant was filtered (0.45 μm PVDF) before injection. Analyses were carried out on an Agilent 1100 HPLC system equipped with a photodiode array detector and an ion-trap mass spectrometer detector in series (Agilent Technologies, Waldbronn, Germany). The separation of phenolic compounds was achieved on a reverse phase LiChroCART C-18 column (Merck, Darmstadt, Germany) (250 × 4 mm, 4.5 μm particle size), operating at room temperature and a flow rate of 0.8 mL/min. A sample volume of 10 μL was injected. The mobile phases used were water with formic acid (1%) (phase A) and acetonitrile (phase B) and the solvent gradient was as follows: 0 min, 1% B, 0 to 10 min, 1%–9% B; 10 to 48 min, 9%–35% B; 48 to 52 min, 35%–95% B; 52 to 54 min, 95%–1% B and maintained at 1 % for 6 min (total run = 60 min). The UV-Vis spectra were acquired in the range of 200 to 600 nm. Compounds were monitored at 260 nm. Nitrogen was used as drying and nebulising gas in the electrospray interface (ESI) with pressure at 65 psi, flow 11 L/min and temperature 350 °C. MS and MS/MS spectra were recorded in negative mode in the range of *m*/*z* 100–1500 with target mass of 700. Identification of compounds was carried out by their spectral properties, molecular mass and literature information. The calibration curves of nuzhenide and GL3 were constructed from 1 ppm to 500 ppm in MeOH/water (50/50, *v*/*v*).

### 3.3. Human Intervention Study

This pilot study was conducted conformed to ethical guidelines outlined in the Declaration of Helsinki and its amendments and was approved by the Research Ethical Committee of the Catholic University of San Antonio (Murcia, Spain). Prior to their participation, the volunteers were informed of the background, objectives, methodology, risks of the intervention, as well as of the type of results and benefits expected and gave written informed consent. Inclusion criteria were as follows: young (20–25 year old) healthy men and women (body mass index, BMI < 30 kg/m^2^), non-smoker and non-alcohol consumers (≤30 g/day), with no-abnormal dietary habits or vitamin supplementation. Participants were excluded if they were taking medication and/or had consumed antibiotics for 3 months prior to the intervention, or if they suffered from any hemostatic or metabolic disturbances, gastrointestinal and/or cardiovascular diseases, or any other chronic disease. The only dietary source known to contain some secoiridoids (mostly oleuropein, ligstroside and hydroxytyrosol [13]) is olives and derived products, such as olive oil. Since it has been reported that olive metabolites are eliminated well within 24 h [13], the volunteers were instructed to avoid all olive products during the three days previous to the study. Volunteers gathered in a room specifically designed for this purpose for the duration of the intervention. A total of nine volunteers completed the study. This sample size is comparable to previous studies in the field [11,13,20,22]. The characteristics of the participants are summarized in Table 4.

**Table 4:** Summary of the demographic and anthropomorphic characteristics of the volunteers taking part in the study. Data are shown as the mean value ± SD.

N	10
Gender (M/F)	(4/6)
Age (years old)	23.5 ± 1.3
Weight (kg)	(M) 81.0 ± 12.0
	(F) 69.1 ± 7.2
BMI (kg/m^2^)	(M) 25.1 ± 2.8
	(F) 21.5 ± 2.1

**Figure 7:**
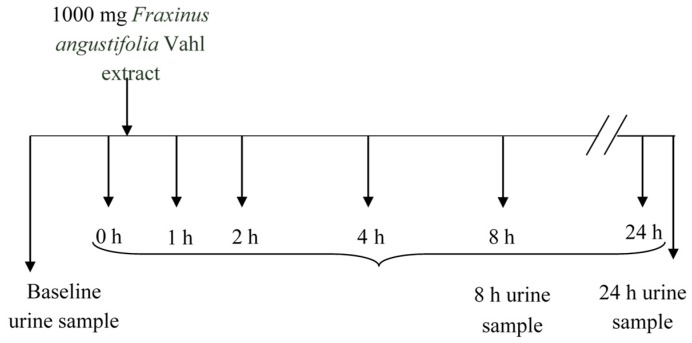
Experimental design and sampling protocol.

The sampling protocol is depicted in Figure 7. Blood (5 mL) was collected into heparinized tubes from the antecubital arm vein: one sample at baseline (8.00 a.m. before *Fraxinus* intake, control) and several samples at predetermined time points (1, 2, 4, and 8 h) following a single acute dose of the *Fraxinus* extract in hard capsules (3 × 334 mg) with a glass of water. This dose was chosen according to that used in previous animal and human studies [2,3,4,5,6,7] reporting some metabolic beneficial effects. The volunteers were asked to come back the following morning (8.00 a.m. before breakfast) for a further 24 h blood sample extraction. Blood samples were centrifuged (3000× *g*, 15 min) and plasma was frozen prior to metabolites analysis. The participants were also given special containers for urine collection. They collected three urine samples: at baseline (taken the morning of the study before *Fraxinus* intake) as well as the urine produced during the following 8 and 24 h after the extract consumption. Total urine volume was measured and all urine samples were frozen until further analysis.

### 3.4. Urine and Plasma Samples Preparation

Urine samples were thawed, rapidly vortexed to homogenize the sample, then centrifuged at 14,000× *g* for 10 min and filtered (0.45 μm PVDF). Prior to injection, the samples were diluted (1:5) with acidified water (0.1% formic acid). Plasma samples were thawed and 200 μL mixed and extracted with 600 μL ACN:formic acid (99.8:0.2, *v*/*v*) by vortexing for 2 min followed by ultrasonic bath for 10 min. The mixture was centrifuged at 14,000× *g* for 10 min and the supernatant was reduced to dryness in a speed vacuum concentrator. The dried samples were re-suspended in 200 μL of MeOH/water (50/50, *v*/*v*) plus 0.1% formic acid and filtered (0.45 μm PVDF filter) prior to analysis. Quercetin (IS) was added just before injection of both types of samples (final concentration 0.5 ppm).

### 3.5. UPLC-ESI-QTOF MS Analysis of Plasma and Urine Samples

The analyses were carried out using an Agilent 1290 Infinity LC system coupled to the 6550 Accurate-Mass QTOF (Agilent Tecnologies, Waldbronn, Germany) with electrospray interface (Jet Stream Technology). Samples were injected (2 μL) on a reverse phase Poroshell 120 EC-C18 column (3 × 100 mm, 2.7 μm) (Agilent Tecnologies) operating at 30 °C and a flow rate of 0.4 mL/min. The mobile phases used were acidified water (0.1% formic acid) (Phase A) and acidified ACN (0.1% formic acid) (Phase B). Compounds were separated using the following gradient conditions: 0–10 min, 1%–18% phase-B; 10–16 min, 18%–38% phase-B; 16–22 min, 38%–95% phase-B. Finally, the phase B content was returned to the initial conditions (1%) in 1 min and the column re-equilibrated for 5 min.

The best conditions for the electrospray interface were: gas temperature 280 °C, drying gas 9 L/min, nebulizer 35 psi, sheath gas temperature 400 °C, sheath gas flow 12 L/min. Spectra were acquired in the range 100–1100 *m*/*z* in negative mode, fragmentor was 100 V and acquisition rate 1.5 spectra/s. Targeted MS/MS analyses were developed to add confidence to compound identification. MS/MS product ion spectra were collected at an *m*/*z* range of 50–800 using a retention time window of 1 min, an isolation window of 4.0 amu, collision energy of 20 V and an acquisition rate of 4 spectra/s. To assure the desired mass accuracy of recorded ions, continuous internal calibration was performed during analyses with the use of signals *m*/*z* 112.9855 (detected *m*/*z* [C_2_O_2_F_3_ − H]^−^) and 1033.9881 (detected *m*/*z* [C_18_H_18_O_6_N_3_P_3_F_24_ + TFA-H]^−^). Data were processed using the Mass Hunter Qualitative Analysis software (version B.06.00, Agilent Technologies). Two strategies were applied for the identification of metabolites: targeted and untargeted analysis.

#### 3.5.1. Targeted Metabolomics Analysis

The target screening strategy consists of searching for a list of predicted compounds after MS full acquisition. The list was created taking into account the information available in the literature about the absorption and metabolism of other secoiridoids [13,16] or other families of phenolic compounds [35]. We first searched for the presence of the compounds originally found in the extracts (mainly secoiridoids glucosides) (Figure 1 and Table 1) as well as possible compounds that may derive from the hydrolysis of these glucosides, especially those that may be released by the action of glycosidases, *i.e.*, tyrosol and ligstroside-aglycone (Figure 1). We then searched for the presence of compounds potentially derived from the metabolic transformation of the parent glucosides and aglycone fragments. We specifically searched for phase I and phase II conjugates (glucuronide, sulfate, sulfoglucuronide, methyl-derivatives and hydroxylated derivatives) of all the compounds. The complete list of the investigated metabolites, molecular formula and exact mass is included in Supplementary Table S1. Screening was based on mass filtering at the exact mass of the metabolite investigated using narrow mass extraction windows (0.01 *m*/*z*). The identification was possible with the support of the information attained in the QTOF-MS acquisition mode that provides potential molecular formulae for the compounds based on the accurate mass and isotopic pattern.

#### 3.5.2. Untargeted Metabolomics Analysis Steps

To explore the potential presence in plasma and urine samples of other non-anticipated metabolites derived from the metabolic transformation of the secoiridoids, we developed and applied an untargeted metabolomics approach. To cope with the complexity of the results obtained by UPLC-QTOF measurements, date pre-processing and statistical analyses were implemented. Initial data processing was carried out using the Molecular Feature Extraction (MFE) algorithm. Ions were extracted as molecular features (MFs) characterized by retention time, intensity of the chromatographic peak and accurate mass. For the extraction procedure (both in plasma and urine samples), the minimum absolute abundance was set at 5000 counts and the minimum number of ions at 2. Further, the *m*/*z* range was between 100 and 1500 and the time range between 0.5 and 20 min. Data files in compound exchange format (.cef files) were created for each sample and exported into the Mass Profiler Professional (MPP) software package (version B.12.01, Agilent Technologies, Santa Clara, CA, USA) for further processing. Alignment of retention time and *m*/*z* values was carried out across the samples using a tolerance window of 0.2 min and 15 ppm of the observed *m*/*z* + 2 mDa. To reduce the dimensionality of the data prior to statistical analyses and to focus on the objective of the analysis, all the MFs that were not present in at least 50% of the samples (volunteers) in at least one group (time-point after the FXE intake) were removed. Only those features present in 5 out of 9 volunteers in at least one time were analyzed. Using one-way ANOVA (*p* < 0.05, Benjamini-Hochberg false discovery rate (FDR) multiple testing correction) and a fold-change cut-off of 2.0 we generated a list of compounds that differed significantly between groups (time-points). The final list of selected ions was subjected to unsupervised principal component analysis (PCA) to visualize differences in metabolite profiles at different times after the intake of FXE. Since this study was mainly focused on the search for metabolites potentially derived from the secoiridoids, all the metabolites with peak intensity higher in the control than after the ingestion (down-regulated) were not considered. Determination of the molecular formula and tentative identification of the rest of compounds (up-regulated) was carried out using accurate mass measurement of full single MS spectra and additional information obtained by MS/MS analyses. These data were compared to those registered in various databases such as METLIN, KEGG LIGAND and Human Metabolome Database (HMD).

## 4. Conclusions

In this study we have applied targeted and non-targeted metabolomic approaches to determine absorbed metabolites following acute ingestion of a *Fraxinus* *angustifolia* seed/fruit extract in human healthy volunteers. LC-QTOF MS analyses of urine and plasma samples revealed limited bioavailability of the intact secoiridoids present in the extract and further metabolism by glycosidases, esterases and phase II conjugation enzymes to form mostly tyrosol and ligstroside-aglycone conjugates. The ligstroside-aglycone conjugates may constitute a good biomarker of exposure to the *Fraxinus* extract. The appearance in plasma and urine of some phenolic derivatives suggest additional metabolism by phase I and (or) microbial enzymes. Further studies are needed to clarify the metabolic conversion of secoiridoids from *Fraxinus* and to advance the knowledge of its metabolic benefits and mechanisms of action.

## Supplementary Materials

Supplementary materials can be accessed at: http://www.mdpi.com/1420-3049/20/12/19845/s1.

## References

[1] Kostova I., Iossifova T. (2007). Chemical components of *Fraxinus* species. Fitoterapia.

[2] Gomez-Garcia F., Flanagan J., García-Molina O., Vilaplana-Vivo V., García-Carrillo N., Fança-Berthon P., Bily A., Roller M., Vicente-Ortega V., Issaly N. (2015). Preventive effect of a *Fraxinus excelsior* L seeds/fruits extract on hepatic steatosis in obese type 2 diabetic mice. J. Diabetes Metab..

[3] Montó F., Arce C., Noguera M.A., Ivorra M.D., Flanagan J., Roller M., Issaly N., D’Ocon P. (2014). Action of an extract from the seeds of *Fraxinus excelsior* L. on metabolic disorders in hypertensive and obese animal models. Food Funct..

[4] Ibarra A., Baia N., Hea K., Bily A., Cases J., Roller M., Sang S. (2011). *Fraxinus excelsior* seed extract FraxiPure™ limits weight gains and hyperglycemia in high-fat diet-induced obese mice. Phytomedicine.

[5] López-Carreras N., Fernández-Vallinas S., Miguel M., Aleixandre A. (2014). Long-term effect of an aqueous *Fraxinus excelsior* L. seed extract in spontaneously hypertensive rats. Int. J. Hypertens..

[6] Visen P., Saraswat B., Visen A., Roller M., Bily A., Mermet C., He K., Bai N., Lemaire B., Lafay S. (2009). Acute effects of *Fraxinus excelsior* L. seed extract on postprandial glycemia and insulin secretion on healthy volunteers. J. Ethnopharmacol..

[7] Zulet M.A., Navas-Carretero S., Lara y Sánchez D., Abete I., Flanagan J., Issaly N., Fanca-Berthon P., Bily A., Martinez J.A. (2014). A *Fraxinus excelsior* L. seeds/fruits extract benefits glucose homeostasis and adiposity related markers in elderly overweight/obese subjects: A longitudinal, randomized, crossover, double-blind, placebo-controlled nutritional intervention study. Phytomedicine.

[8] Flanagan J., Meyer M., Pasamar M.A., Ibarra A., Roller M., Alvarez i Genoher N., Leiva S., Gómez-García F., Alcaraz M., Martínez-Carrasco A. (2013). Safety evaluation and nutritional composition of a *Fraxinus*
*excelsior* seed extract, FraxiPure™. Food Chem. Toxicol..

[9] Bai N., He K., Ibarra A., Bily A., Roller M., Chen X., Rühl R. (2010). Iridoids from *Fraxinus excelsior* with adipocyte differentiation-inhibitory and PPARα activation activity. J. Nat. Prod..

[10] Suárez M., Valls R.M., Romero M.P., Maciá A., Fernández S., Giralt M., Solá R., Motilva M.J. (2011). Bioavailability of phenols from a phenol-enriched olive oil. Br. J. Nutr..

[11] García-Villalba R., Carrasco-Pancorbo A., Nevedomskaya E., Mayboroda O.A., Deelder A.M., Segura-Carretero A., Fernández-Gutiérrez A. (2010). Exploratory analysis of human urine by LC-ESI-TOF MS after high intake of olive oil: Understanding the metabolism of polyphenols. Anal. Bioanal. Chem..

[12] Pinto J., Paiva-Martins F., Corona G., Debnam E.S., Oruna-Concha M.J., Vauzour D., Gordon M.H., Spencer J.P.E. (2011). Absorption and metabolism of olive oil secoiridoids in the small intestine. Br. J. Nutr..

[13] De Bock M., Thorstensen E.B., Derraik J.G.B., Henderson H.V., Hofman P.L., Cutfield W.S. (2013). Human absorption and metabolism of oleuropein and hydroxytyrosol ingested as olive (*Olea europaea* L.) leaf extract. Mol. Nutr. Food Res..

[14] Lockyer S., Corona G., Yaqoob P., Spencer J.P.E., Rowland I. (2015). Secoiridoids delivered as olive leaf extract induce acute improvements in human vascular function and reduction of an inflammatory cytokine: A randomized, double-blind, placebo-controlled, cross-over trial. Br. J. Nutr..

[15] Kendall M., Batterham M., Callahan D.L., Jardine D., Prenzler P.D., Robards K., Ryan D. (2012). Randomized controlled study of the urinary excretion of biophenols following acute and chronic intake of olive leaf supplements. Food Chem..

[16] García-Villalba R., Larrosa M., Possemiers S., Tomás-Barberán F.A., Espín J.C. (2014). Bioavailability of phenolics from an oleuropein-rich olive (*Olea europaea*) leaf extract and its acute effect on plasma antioxidant status: Comparison between pre- and postmenopausal women. Eur. J. Nutr..

[17] Kim S., Kim J., Yun E.J., Kim K.H. (2016). Food metabolomics: From farm to human. Curr. Opin. Biotechnol..

[18] Primrose S., Draper J., Elsom R., Kirkpatrick V., Mathers J.C., Seal C., Beckmann M., Haldar S., Beattie J.H., Lodge J.K. (2011). Metabolomics and human nutrition. Br. J. Nutr..

[19] Nuñez-Sanchez M.A., García-Villalba R., Monedero-Saiz T., García-Talavera N.V., Gómez-Sánchez M.B., Sánchez-Álvarez C., García-Albert A.M., Rodríguez-Gil F.J., Ruiz-Marín M., Pastor-Quirante F.A. (2014). Targeted metabolic profiling of pomegranate polyphenols and urolithins in plasma, urine and colon tissues from colorectal cancer patients. Mol. Nutr. Food Res..

[20] Kristensen M., Engelsen S.B., Dragsted L.O. (2012). LC-MS metabolomics top-down approach reveals new exposure and effect biomarkers of apple and apple-pectin intake. Metabolomics.

[21] Xie G., Zhao A., Zhao L., Chen T., Chen H., Qi X., Zheng X., Ni Y., Cheng Y., Lan K. (2012). Metabolic fate of tea polyphenols in humans. J. Proteome Res..

[22] Llorach R., Garrido I., Monagas M., Urpi-Sarda M., Tulipani S., Bartolome B., Andres-Lacueva C. (2010). Metabolomics study of human urinary metabolome modifications after intake of almond (*Prunus dulcis* (Mill.) D.A. Webb) skin polyphenols. J. Proteome Res..

[23] Silva S., Gomes L., Leitao F., Bronze M., Coelho A.V., Vilas-Boas L. (2010). Secoirioids in olive seed: Characterization of nuzhenide and 11-methyl oleosides by liquid chromatography with diode array and mass spectrometry. Grasas Aceites.

[24] Del Boccio P., di Deo A., de Curtis A., Celli N., Iacoviello L., Rotilio D. (2003). Liquid chromatography-tandem mass spectrometry analysis of oleuropein and its metabolite hydroxytyrosol in rat plasma and urine after oral administration. J. Chromatogr. B.

[25] Guo N., Zhu M., Han X., Sui D., Wang Y., Yang Q. (2014). The metabolism of salidroside to its aglycone *p*-tyrosol in rats following the administration of salidroside. PLoS ONE.

[26] Akao T., Kobashi K., Aburada M. (1994). Enzymic studies on the animal and intestinal bacterial metabolism of geniposide. Biol. Pharm. Bull..

[27] Rodriguez-Mateos A., Cifuentes-Gomez T., Gonzalez-Salvador I., Ottaviani J.I., Schroeter H., Kelm M., Heiss C., Spencer J. (2015). Influence of age on the absorption, metabolism, and excretion of cocoa flavanols in healthy subjects. Mol. Nutr. Food Res..

[28] García-Conesa M.T. (2015). Dietary polyphenols against metabolic disorders: How far have we progressed in the understanding of the molecular mechanisms of action of these compounds?. Crit. Rev. Food Sci. Nutr..

[29] Mosele J.I., Martín-Peláez S., Maciá A., Farrás M., Valls R.M., Catalán U., Motilva M.J. (2014). Faecal microbial metabolism of olive oil phenolic compounds: *In vitro* and *in vivo* approaches. Mol. Nutr. Food Res..

[30] D’Angelo S., Manna C., Migliardi V., Mazzoni O., Morrica P., Capasso G., Pontoni G., Galleti P., Zappia V. (2001). Pharmacokinetics and metabolism of hydroxytyrosol, a natural antioxidant from olive oil. Drug Metab. Dispos..

[31] Chandramohan R., Pari L., Rathinam A., Ahmad Sheikh B. (2015). Tyrosol, a phenolic compound, ameliorates hyperglycemia by regulating key enzymes of carbohydrate metabolism in streptozotocin induced diabetic rats. Chem. Biol. Interact..

[32] Bohn T. (2014). Dietary factors affecting polyphenol bioavailability. Nutr. Rev..

[33] Romo-Vaquero M., Selma M.V., Larrosa M., Obiol M., García-Villalba R., González-Barrio R., Issaly N., Flanagan J., Roller M., Tomás-Barberán F.A. (2014). A rosemary extract rich in carnosic acid selectively modulates caecum microbiota and inhibits β-glucosidase activity, altering fiber and short chain fatty acids fecal excretion in lean and obese female rats. PLoS ONE.

[34] He K., Roller M., Bily A., Bai N., Dikansky J., Ibarra A. (2012). Extract of Fraxinus Excelsior Seeds and Therapeutic Applications Therefor.

[35] Del Rio D., Rodriguez-Mateos A., Spencer J.P.E., Tognolini M., Borges G., Crozier A. (2013). Dietary (poly) phenolics in human health: Structures, bioavailability, and evidence of protective effects against chronic diseases. Antioxid. Redox Signal.

