# Supplementary material for: Targeted and Untargeted Metabolomics to Explore the Bioavailability of the Secoiridoids from a Seed/Fruit Extract (Fraxinus angustifolia Vahl) in Human Healthy Volunteers: A Preliminary Study
Source: Molecules. 2015 Dec 11;20(12):22202–19. doi: 10.3390/molecules201219845 (PMC6332458; doi:10.3390/molecules201219845)
Supplement: Supplementary file 1 [file molecules-20-19845-s001.pdf]

# Targeted and Untargeted Metabolomics to Explore the Bioavailability of the Secoiridoids from a Seed/Fruit Extract (*Fraxinus angustifolia* Vahl) in Human Healthy Volunteers: A Preliminary Study

**Rocío García-Villalba, Francisco A. Tomás-Barberán, Pascale Fança-Berthon, Marc Roller, Pilar Zafrilla, Nicolas Issaly and Maria-Teresa García-Conesa**

**Table S1.** Targeted metabolomics analysis: potential compounds specifically derived from the metabolic transformation of the secoiridoid glucosides identified in the *Fraxinus angustifolia* Vahl extract.

| **Parent Compounds** | | | **Aglycone Fragments** | | |
|---|---|---|---|---|---|
| Potential Metabolite | Molecular formula | Exact Mass | Potential Metabolite | Molecular formula | Exact Mass |
| Nuzhenide/ Excelside B/1-O-β-D-glucosylformoside * | $C_{31}H_{42}O_{17}$ | 685.2349 | Tyrosol | $C_8H_{10}O_2$ | 137.0608 |
| + glucuronide | $C_{37}H_{50}O_{23}$ | 861.2670 | + glucuronide | $C_{14}H_{18}O_8$ | 313.0929 |
| + sulfate | $C_{31}H_{42}O_{20}S$ | 765.1917 | + sulfate | $C_8H_{10}O_5S$ | 217.0176 |
| + sulfoglucuronide | $C_{37}H_{50}O_{26}S$ | 941.2238 | + sulfoglucuronide | $C_{14}H_{18}O_{11}S$ | 393.0497 |
| + methyl | $C_{38}H_{52}O_{26}S$ | 955.2395 | + methyl | $C_9H_{12}O_2$ | 151.0765 |
| GL3/nuzhenide 11-methyl oleoside * | $C_{48}H_{64}O_{27}$ | 1071.3562 | Hydroxytyrosol (Tyrosol+OH) | $C_8H_{10}O_3$ | 153.0557 |
| + glucuronide | $C_{54}H_{72}O_{33}$ | 1247.3883 | + glucuronide | $C_{14}H_{18}O_9$ | 329.0878 |
| + sulfate | $C_{48}H_{64}O_{30}S$ | 1151.3130 | + sulfate | $C_8H_{10}O_6S$ | 233.0125 |
| + sulfoglucuronide | $C_{54}H_{72}O_{36}S$ | 1327.3451 | + sulfoglucuronide | $C_{14}H_{18}O_{12}S$ | 409.0446 |
| + methyl | $C_{49}H_{66}O_{27}$ | 1085.3719 | + methyl | $C_9H_{12}O_3$ | 167.0714 |
| GL5 | $C_{42}H_{54}O_{22}$ | 909.3034 | Ligstroside-aglycone | $C_{19}H_{22}O_7$ | 361.1293 |
| + glucuronide | $C_{48}H_{62}O_{28}$ | 1085.3355 | + glucuronide | $C_{25}H_{30}O_{13}$ | 537.1614 |
| + sulfate | $C_{42}H_{54}O_{25}S$ | 989.3602 | + sulfate | $C_{19}H_{22}O_{10}S$ | 441.0861 |
| + sulfoglucuronide | $C_{48}H_{62}O_{31}S$ | 1165.2923 | + sulfoglucuronide | $C_{25}H_{30}O_{16}S$ | 617.1182 |
| + methyl | $C_{43}H_{56}O_{22}$ | 923.319 | + methyl | $C_{20}H_{24}O_7$ | 375.1449 |
| Salidroside (Tyrosol glucose) | $C_{14}H_{20}O_7$ | 299.1136 | Oleuropein aglycone (Ligstroside-aglycone + OH) | $C_{19}H_{22}O_8$ | 377.1242 |
| + glucuronide | $C_{20}H_{28}O_{13}$ | 475.1457 | + glucuronide | $C_{25}H_{30}O_{14}$ | 553.1563 |
| + sulfate | $C_{14}H_{20}O_{10}S$ | 379.0704 | + sulfate | $C_{19}H_{22}O_{11}S$ | 457.0810 |
| + sulfoglucuronide | $C_{20}H_{28}O_{16}S$ | 555.1025 | + sulfoglucuronide | $C_{25}H_{30}O_{17}S$ | 633.1131 |
| + methyl | $C_{15}H_{22}O_7$ | 313.1293 | + methyl | $C_{20}H_{24}O_8$ | 391.1398 |
| Oleoside | $C_{16}H_{22}O_{11}$ | 389.1089 | Elenolic acid | $C_{11}H_{14}O_6$ | 241.0718 |
| + glucuronide | $C_{22}H_{30}O_{17}$ | 565.1410 | + glucuronide | $C_{17}H_{22}O_{12}$ | 417.1038 |
| + sulfate | $C_{16}H_{22}O_{14}S$ | 469.0657 | + sulfate | $C_{11}H_{14}O_9S$ | 321.0286 |

**Table S1.** *Cont.*

| Parent Compounds | | | Aglycone Fragments | | |
|---|---|---|---|---|---|
| + sulfoglucuronide | $C_{22}H_{30}O_{20}S$ | 645.0978 | + sulfoglucuronide | $C_{17}H_{22}O_{15}S$ | 497.0607 |
| + methyl | $C_{17}H_{24}O_{11}$ | 403.1246 | + methyl | $C_{12}H_{16}O_{6}$ | 255.0874 |
| Elenolic acid glucose (Oleoside-11 methyl ester) | $C_{17}H_{24}O_{11}$ | 403.1246 | Demethyl-elenolic acid | $C_{10}H_{12}O_{6}$ | 227.0561 |
| +glucuronide | $C_{23}H_{32}O_{17}$ | 579.1567 | + glucuronide | $C_{16}H_{20}O_{12}$ | 403.0882 |
| +sulfate | $C_{17}H_{24}O_{14}S$ | 483.0814 | + sulfate | $C_{10}H_{12}O_{9}S$ | 307.0129 |
| +sulfoglucuronide | $C_{23}H_{32}O_{20}S$ | 659.1135 | + sulfoglucuronide | $C_{16}H_{20}O_{15}S$ | 483.045 |
| + methyl | $C_{18}H_{26}O_{11}$ | 417.1402 | | | |
| Ligstroside | $C_{25}H_{32}O_{12}$ | 523.1821 | Elenolic-Tyrosol-Elenolic | $C_{30}H_{34}O_{12}$ | 585.1978 |
| + glucuronide | $C_{31}H_{40}O_{18}$ | 699.2142 | + glucuronide | $C_{36}H_{42}O_{18}$ | 761.2298 |
| + sulfate | $C_{25}H_{32}O_{15}S$ | 603.2142 | + sulfate | $C_{30}H_{34}O_{15}S$ | 665.1546 |
| + sulfoglucuronide | $C_{31}H_{40}O_{21}S$ | 779.1710 | + sulfoglucuronide | $C_{36}H_{42}O_{21}S$ | 841.1867 |
| + methyl | $C_{26}H_{34}O_{12}$ | 537.1978 | + methyl | $C_{31}H_{36}O_{12}$ | 599.2134 |

*: isomers with the same molecular formulae.
